# Capturing Rest-Activity Profiles in Schizophrenia Using Wearable and Mobile Technologies: Development, Implementation, Feasibility, and Acceptability of a Remote Monitoring Platform

**DOI:** 10.2196/mhealth.8292

**Published:** 2018-10-30

**Authors:** Nicholas Meyer, Maximilian Kerz, Amos Folarin, Dan W Joyce, Richard Jackson, Chris Karr, Richard Dobson, James MacCabe

**Affiliations:** 1 Department of Psychosis Studies Institute of Psychiatry, Psychology and Neuroscience King's College London London United Kingdom; 2 South London and Maudsley National Health Service Foundation Trust Bethlem Royal Hospital Beckenham, Kent United Kingdom; 3 Department of Biostatistics and Health Informatics Institute of Psychiatry, Psychology and Neuroscience King's College London London United Kingdom; 4 Audacious Software Chicago, IL United States; 5 Center for Behavioural Intervention Technologies Northwestern University Chicago, IL United States

**Keywords:** sleep, circadian rhythm, mHealth, smartphone, relapse, psychosis

## Abstract

**Background:**

There is growing interest in the potential for wearable and mobile devices to deliver clinically relevant information in real-world contexts. However, there is limited information on their acceptability and barriers to long-term use in people living with psychosis.

**Objective:**

This study aimed to describe the development, implementation, feasibility, acceptability, and user experiences of the *Sleepsight* platform, which harnesses consumer wearable devices and smartphones for the passive and unobtrusive capture of sleep and rest-activity profiles in people with schizophrenia living in their homes.

**Methods:**

A total of 15 outpatients with a diagnosis of schizophrenia used a consumer wrist-worn device and smartphone to continuously and remotely gather rest-activity profiles over 2 months. Once-daily sleep and self-rated symptom diaries were also collected via a smartphone app. Adherence with the devices and smartphone app, end-of-study user experiences, and agreement between subjective and objective sleep measures were analyzed. Thresholds for acceptability were set at a wear time or diary response rate of 70% or greater.

**Results:**

Overall, 14 out of 15 participants completed the study. In individuals with a mild to moderate symptom severity at baseline (mean total Positive and Negative Syndrome Scale score 58.4 [SD 14.4]), we demonstrated high rates of engagement with the wearable device (all participants meeting acceptability criteria), sleep diary, and symptom diary (93% and 86% meeting criteria, respectively), with negative symptoms being associated with lower diary completion rate. The end-of-study usability and acceptability questionnaire and qualitative analysis identified facilitators and barriers to long-term use, and paranoia with study devices was not a significant barrier to engagement. Comparison between sleep diary and wearable estimated sleep times showed good correspondence (ρ=0.50, *P*<.001).

**Conclusions:**

Extended use of wearable and mobile technologies are acceptable to people with schizophrenia living in a community setting. In the future, these technologies may allow predictive, objective markers of clinical status, including early markers of impending relapse.

## Introduction

### Background

Approximately 80% of those treated for the first episode of psychosis experience at least 1 further episode within 5 years [1]. Relapse often goes undetected until the individual is severely unwell, by which time the episode is disabling, distressing, and compromises illness trajectory [2]. Developing systems for detecting the early signs of deterioration [3] and prompting preventative interventions that avert relapse and hospital admission is, therefore, a priority.

Disturbances in rest-activity patterns are frequently reported in the early stages of relapse in schizophrenia. Sleep disturbance is the most common relapse indicator to be identified by family members [4], and it is reported by 68% [5] to 79% [6] of patients. They commonly precede relapse by over a week [5], suggesting they occur in the early stages of deterioration. Insomnia, sleep fragmentation, and reduced total sleep time have been associated with severity of psychotic experiences [7-9], and recent evidence suggests that sleep disruption may play a causal role in the genesis of psychotic symptoms [10,11]. Similarly, the organization of motor activity has been shown to vary with the severity of psychotic symptoms [12,13]. Sleep and rest-activity disturbance, therefore, appear to be an important, objective early sign of deterioration in clinical status.

Rest-activity profiles across phases of illness are poorly characterized, in part due to the challenges of sampling these rhythms over long periods in individuals with psychosis. To date, objective estimation of sleep under free-living conditions has been derived from wrist actigraphy [14,15], which estimates sleep parameters from patterns of rest-activity [16]. Actigraphy typically lacks wireless capability, thereby necessitating home or clinic visits for the manual download of data and precluding the real-time data upload necessary for triggering preventative interventions. Actigraphs are recognizable as clinical devices, which can be stigmatizing and aesthetically unacceptable to the user [17] and limits their suitability for apps where long-term use in naturalistic settings is required.

### The Case for Wearable and Mobile Technologies

Recent advances in consumer wearable technology offer several advantages over actigraphy for the remote, continuous, and unobtrusive sampling of rest-activity patterns across phases of illness, in ecologically valid settings. Marketed as lifestyle devices for improving health and well-being, they are designed to be aesthetically and functionally appealing for everyday use, and might, therefore, be more compatible with extended use than currently available actigraphy. Worn on the wrist, such devices measure motor activity via accelerometry and some sample mean heart rate using photoplethysmography. Heart rate may provide an additional predictive signal as cardiovascular regulation also expresses a circadian oscillation [18], and increased mean heart rate [19] and reduced variability [20,21] have been associated with increasing severity of psychotic symptoms.

Crucially, most devices now communicate automatically and wirelessly with smartphones, thereby reducing the burden of data collection by allowing information to be uploaded over the mobile network and delivered to a researcher, clinician, or patient in near real-time. Some manufacturers provide an application programming interface (API), allowing researchers and software developers to access data directly from the company’s servers. The average cost of consumer wearable devices is a quarter to a third of that of currently available research actigraphs.

Accelerometers, gyroscopes, ambient light, and other sensors within smartphones can also be harnessed to infer rest-activity patterns through the *passive* sensing of motor activity, geographical location, and phone usage, among other variables [22,23]. Touchscreen technology facilitates the *active* capture of self-rated measures of symptomatology [24] as well as the delivery of psychological interventions [25] and information for promoting self-management [26]. Smartphone ownership and mobile data coverage continue to expand in developing and advanced economies [27], and mobile phone ownership has increased among people living with mental illness [28,29]. mHealth approaches that employ wearable and smartphone technologies, therefore, show promise as innovative, cost-effective, and scalable “4P interventions” [30]—predictive, pre-emptive, personalized, and participatory—in the management of mental disorders in real-world settings.

Although several studies have explored the feasibility and acceptability of smartphone interventions in schizophrenia [24,26,28,31], wearable devices have received less attention. Studies using traditional actigraphy in schizophrenia have generally lasted in the order of several hours to days [15] to at most 28 days [7]. With regard to consumer devices, 1 study found good levels of adherence with a clip-on activity tracking device over a 6-month period in individuals with a range of psychiatric diagnoses, 8 of whom had schizophrenia-spectrum diagnoses [32]. However, these findings were in the context of promoting weight loss and physical activity and did not examine 24-hour adherence. Whether the longer-term, continuous use of wearable devices is acceptable to individuals with schizophrenia has not been studied. Schizophrenia is associated with paranoid beliefs and impairments in motivation and cognition, which may interfere with engagement with mobile and wearable technologies. Considerable challenges surrounding device validation and the regulatory and ethical frameworks for employing consumer devices in clinical contexts also exist [33].

**Figure 1:**
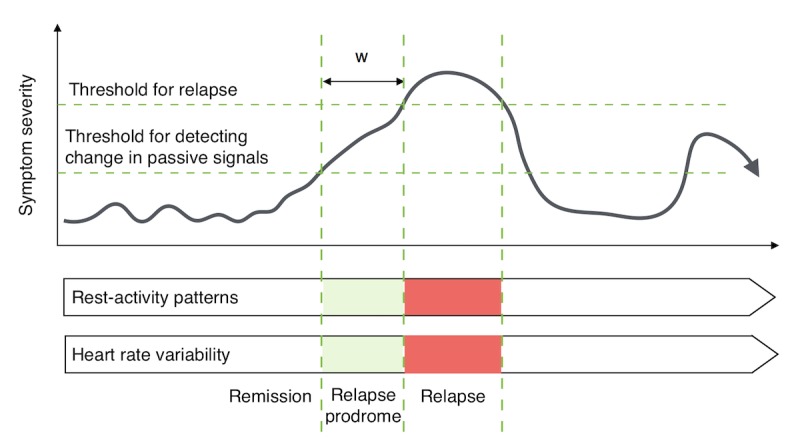
A theoretical overview of the approach, where continuous passive variables that have been shown to co-vary with severity of psychopathology, including rest-activity profiles and heart rate variability, are captured using digital technologies (bottom). Disturbances in these variables may be detectable in the early stages of relapse (top), thus providing a window for preventative intervention (w).

### The *Sleepsight* Study

This pilot study represents the first stage in an ongoing program of research for applying wearable and smartphone technologies for the identification of early signs of deterioration in schizophrenia, with a particular emphasis on passive sensing of rest-activity rhythms (Figure 1). The purpose of this paper is (1) to describe the user-centered development and implementation of a platform for sampling rest-activity profiles in schizophrenia; (2) to evaluate the feasibility, acceptability, and user experience of the system in outpatients with schizophrenia over an 8-week period; and (3) to provide preliminary evidence demonstrating that meaningful passive behavioral parameters and self-rated subjective data can be captured using the platform. The mHealth Evidence and Assessment checklist [34,35] developed through World Health Organization expert consensus for improving the generalizability and replicability of mHealth research was used as a framework for synthesizing and reporting this work.

## Methods

### Principles of Platform Development

The *Sleepsight* platform was developed through collaboration between patients, clinicians, bioinformaticians, and software developers, and it was guided by the following core principles:

1. User-centered design: People living with psychosis were involved throughout the development and testing cycle. Consultation groups consisted of patients who advised on the selection of wearable and mobile devices, the design of the software app, and aspects of the study design including recruitment strategy, feedback of data, and incentivization. The subsequent acceptability-feasibility study comprised a separate group of patients living in the community.
2. Integration with everyday life: Initial user-group testing with a range of currently available research wearables (GENEActiv, Activinsights, Cambridge, UK; ActiGraph GT9X Link, Actigraph corp, Pensacola, USA; Empatica E4, Milan, Italy) suggested that they would not be acceptable for extended use due to their design and limited functionality. To enhance acceptability and minimize user burden and stigma, widely available consumer-oriented technologies were therefore considered. The user groups favored the wrist-worn Fitbit Charge HR (Fitbit Inc, San Francisco) due to its appearance as a lifestyle device that is acceptable to both younger and older users and the ability to view metrics relating to sleep and activity via the Fitbit app.
3. Wireless functionality: The Fitbit provided wireless data transfer to a smartphone and allowed access to minute-level activity, sleep, and heart rate data via API calls to the Fitbit server [36]. As with all consumer devices, data are preprocessed on the device to minimize the volume of data transfer and maximize battery life, which precludes access to raw sensor data. The Fitbit required charging approximately every 5 days, taking around 2 hours, and was splashproof but not waterproof. An Android-based Motorola Moto G second-generation smartphone was selected due to its robust build quality, long battery life, relatively low cost, and easy-to-read 5-inch screen.
4. Remote and real-time: Each participant was provided with a 4G mobile data plan, which allowed data to be continuously uploaded to the research server and adherence with the wearable device, sleep, and symptom diary to be monitored in real-time. Participants with a home wireless network could also upload data via this route.
5. Secure: Safeguarding privacy and data security was a cornerstone of the platform. Usernames were obfuscated using an MD5 hash algorithm [37], and data were encrypted and transmitted to the secure end point via the mobile network or participants’ home wireless network. Data were cached on the phone until connectivity was available and cleared from the device following transmission. All participants received a unique identifier, and no personally identifiable digital information was stored or transmitted.
6. Open source software: Code for the platform architecture is publicly available to promote replicability, refinement of the software, and assessment of external validity through studies in other centers.

### The *Sleepsight* Platform

**Figure 2:**
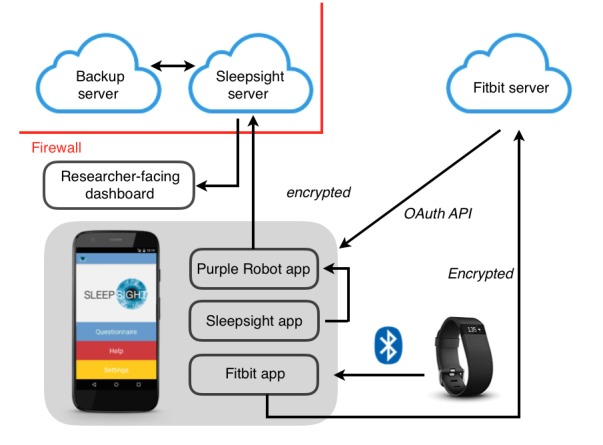
*Sleepsight* platform architecture.

**Figure 3:**
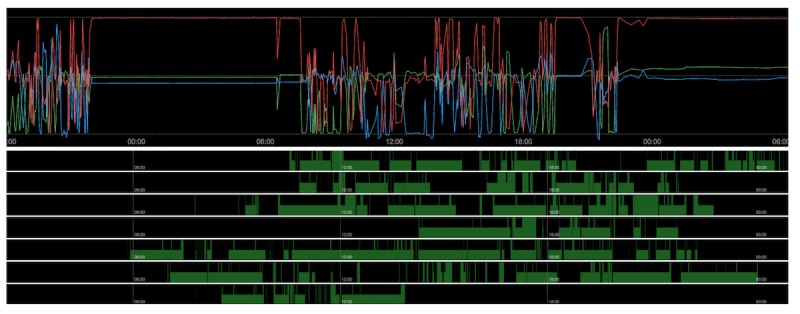
Two sample variables from the researcher-facing dashboard for a single participant. Upper panel: accelerometer output from smartphone showing periods of rest-activity over a continuous 36-hour period; lower panel: smartphone screen state showing screen doze state (lower bars) and active screen state (upper bars) over 7 days.

*Sleepsight* was developed around the Purple Robot [38-40] mobile app (Centre for Behavioral Intervention Technologies, Northwestern University, Chicago, United States), which allows real-time data acquisition from a range of Android smartphone sensors and integrates with wearable devices (Figure 2). Purple Robot operates in the background, with no intervention from the user, and it was configured to sample the smartphone accelerometer and light sensors and access information about device battery level and the frequency of screen-unlock events (Table 1). Global Positioning System (GPS) location, call, or text message content were not captured in response to advice from the user group that this would be perceived as intrusive for many users. Purple Robot consolidated data from 2 further sources: the wearable device—via API calls to the Fitbit server multiple times a day—and the *Sleepsight* app. Data are uploaded to the researcher-facing dashboard, where circadian patterns in rest-activity variables can be visualized in real-time (Figure 3), and interruptions in the data stream are identified and acted upon.

The *Sleepsight* app [41] prompted the user, once a day at a time that was previously agreed with the study team, to complete a 30-second sleep diary (time to bed, time out of bed, and sleep quality) followed by a self-report symptom diary taking 2-3 min to complete. The purpose of the symptom diary was to provide an outcome measure of clinical status against which associations with passive sensor variables could be examined. Each complete submission was followed by a short motivational message (eg, “good job - see you tomorrow!”). The app also provided a help section with instructions on how to use the devices and contact information in case of difficulties.

Interoperability with existing local electronic patient clinical record systems was not supported at this stage, though future integration is planned.

**Table 1:** Passive variables provided by the *Sleepsight* system.

Device and sensor(s)		Output from sensor(s)
**Wearable device**		
Tri-axial accelerometer		Raw data not available^a^
Photoplethysmogram (optical sensor)		Mean heart rate/minute
**Smartphone**		
	Tri-axial accelerometer	Acceleration/g sampled at 10 Hz
	Light sensor	Ambient light intensity/lux
	Battery probe	Battery level and charging events
	Screen event probe	Whether or not the screen was active

^a^Instead, several derived variables computed by Fitbit available: steps per minute; lightly, moderately, and very active minutes; sleep onset, offset, number of awakenings, and total sleep time.

### Participants

A total of 15 adults with a diagnosis of schizophrenia were recruited through clinical teams from community psychiatric services in South London (Table 2). Inclusion criteria were ICD-10 criteria for schizophrenia, aged between 18 and 65 years, and capacity to consent to research; the criteria for exclusion were gross cognitive, sensory, or motor impairments that precluded the use of study devices. Participants were not selected on the basis of clinical status. Most had low-mild symptom intensity at baseline, as reflected in the total Positive and Negative Syndrome Scale (PANSS) scores; however, 2 participants were experiencing significant ongoing psychotic symptoms, with a PANSS positive subscale score above 20 (moderate intensity or greater on at least 5 of the 7 items). Overall, 8 participants had negative symptoms subscale scores of at least 20 (moderate intensity or greater on 5 of the 7 items) and 2 greater than 25 (severe intensity or greater on at least 5 items). All were receiving antipsychotic treatment, and 7 of the participants were prescribed clozapine, as indicated in the United Kingdom for psychosis that is resistant to other antipsychotics. A total of 8 participants lived alone and 7 lived with a spouse, children, or wider family. None of the participants were in regular paid employment. All participants owned a mobile device of their own, and just over half of the participants had a touchscreen device.

### Study Procedures

Screening for eligibility and all clinical assessments were undertaken by an experienced psychiatrist (NM). Participants were asked to use the system continuously for 8 weeks, during this period, passive sensing and symptom rating variables were collected remotely.

On enrollment, informed consent and psychiatric, medical, and sleep histories were obtained. The PANSS, Pittsburgh Sleep Quality Index [42], Insomnia Severity Index [43], and Morningness-Eveningness Questionnaire [44] measures were completed. Participants were provided with the study devices and cables and adapters for charging, and a 4G mobile contract with 5 GB data allowance per month was also provided. Participants received an individual 45-min face-to-face training session, where the use of the smartphone, *Sleepsight* app, wearable device, and its synchronization were explained.

**Table 2:** Clinical characteristics of participants at baseline.

Clinical characteristics		Statistics
**Sex, n (%)**		
	Male	9 (60)
	Female	6 (40)
Age, mean (range)		44.1 (30-54)
Duration of illness in years, mean (range)		16.6 (5-33)
**Mean Positive and Negative Syndrome Scale, (range, SD)**		
	Positive subscale	13.5 (7-23; 4.3)
	Negative subscale	19.7 (8-36; 7.5)
	General subscale	25.1 (16-40, 6.4)
	Total score	58.4 (32-81; 14.4)
**Medication, n (%)**		
	Clozapine	7 (47)
	Oral antipsychotic	5 (33)
	Depot antipsychotic	3 (20)
**Mobile device ownership, n (%)**		
	Nontouchscreen mobile phone	7 (47)
	Touchscreen smartphone	8 (53)

They were asked to wear the Fitbit on the nondominant wrist at all times, except for when bathing or swimming, and they were advised to charge the smartphone once a day when going to bed and to charge the device every 4 to 5 days. It was explained that the purpose of the study was to assess feasibility and acceptability; therefore, putative markers of relapse would not be monitored or responded to during the study.

In addition to the provision of a mobile data plan, it was explained to participants on enrollment that they would be given the option of keeping the wearable device and smartphone on study completion to provide an incentive for engagement and completion and compensation for their time. However, the equipment would remain the property of the research team for the duration of the study, and they would be asked to return them if they withdrew before study completion. This strategy was formulated with user groups, and it was thought to be a fair and appropriate approach.

The 8-week data collection period then began, and participants were free to use the smartphone for making calls, messages, and Web browsing. Participants were asked to use the study smartphone as their primary device, and where necessary, their pre-existing telephone number and personal information were transferred to the new device. Participants received a personalized encouragement text message once weekly, with the aim of promoting motivation and engagement with the research team, and participants were instructed not to perform any updates to the Fitbit firmware during the course of their participation. Data from each user were monitored on a daily basis. Where there was evidence of continued nonadherence for 2 or more days—for example, no signal from the wearable device—a text message prompt was sent to the participant, followed by telephone contact if necessary.

### Outcome Measures

Feasibility was defined as the proportion of participants using each element of the system (wearable device, sleep log, and symptom log) for at least 70% of the 8-week study period. “Definitely feasible” was defined as ≥70% participants meeting the criteria; “possibly feasible” as 50% to 69% participants; and “not feasible” as <50% of participants. Removal of the wearable device was detected by the absence of the heart rate signal. In a test conducted separately (see Multimedia Appendix 1), absence of heart rate signal was found to be a highly valid proxy for nonwear.

The symptom diary items were developed and validated in schizophrenia in the Clintouch study [24] against the PANSS [45], and it consisted of items relating to mood, anxiety, hallucinations, grandiosity, and paranoia.

Acceptability and usability were assessed at the end of the study using a questionnaire conducted in person, adapted from previous studies [26] (Multimedia Appendix 1). Subsequent discussion of user experiences arising from the questionnaire was transcribed verbatim during the interview to further explore attitudes and individual experiences toward the technology.

### Data Analysis

Quantitative analyses were undertaken using R software [46]. Associations between clinical parameters at baseline (PANSS positive score, PANSS negative score, and age) and adherence to wearable device, sleep, and symptom diaries were calculated using Spearman correlation coefficients for nonparametric data.

End-of-study questionnaires are reported descriptively. Interview transcripts were cross-checked for accuracy by a second researcher. Analysis of qualitative end-of-study user experiences followed a grounded theory approach [45], using a coding frame based on major and minor categories emerging from the data, which were refined through discussion between 2 research clinicians.

Ethical approval was obtained from the Dulwich Research and Ethics Committee, London, United Kingdom. As a research study that did not alter routine clinical care and had no intention of commercialization, national regulatory approval from the Medicines and Healthcare Regulatory Agency was not required at this stage.

## Results

### Adherence and Feasibility

Of the 15 participants, 14 completed the study, with varying levels of adherence to each element of the system. One participant withdrew from the study at the end of week 1 due to finding the wearable device uncomfortable, particularly in bed, and returned the study devices. That participant did not report suspicion toward the devices as a cause of discontinuation and had a low total PANSS score of 47. Rather than adopting the study phone, 1 participant wished to continue to use her own smartphone, and it was therefore agreed that she would use this in parallel with the study device. The mean monthly data volume generated by the system per participant was approximately 1.1 GB, not including personal data use. One participant used the monthly data allocation within the first 3 weeks and was provided with an unlimited data plan for the remainder of the study. There was no loss or damage to devices.

For the wearable device, all the participants exceeded the 70% threshold for feasibility, with a mean wear time of 21.8 hours/day or 91% of the total study duration.

**Figure 4:**
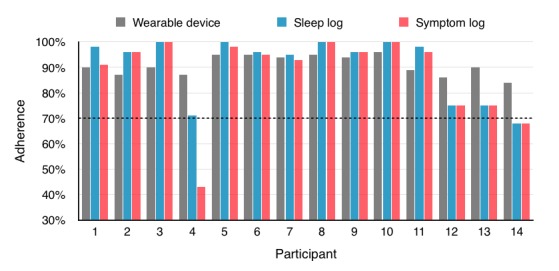
Overall adherence to wearable device, sleep, and symptom diaries for each participant.

For the *Sleepsight* app, 93% (13/14) of participants met the feasibility criteria for completion of the daily sleep diary, with a mean average of 91% (51/56) of all possible questionnaires being completed. Moreover, 86% (12/14) of participants met the feasibility criteria for completion of the symptom diary, with a mean average of 88% (49/56) of all possible questionnaires being filled (Figure 4). Participants 4 and 14, whose sleep and symptom diary completion rates were at or below the feasibility threshold, scored 29 and 36, respectively, on the negative symptoms scale of the PANSS, consistent with negative symptoms in the severe range, and both had a history of treatment-resistant illness. Participant 4 received 4 further weekly top-up training sessions after reporting difficulties in completing the diaries and using the smartphone for making calls and sending text messages.

Spearman correlation demonstrated a significant negative relationship between PANSS positive score and sleep diary completion (ρ=−.49, *P*<.05) and symptom diary completion (ρ=−.40, *P*<.01). PANSS negative score was negatively correlated with wearable adherence (ρ=−.49, *P*<.05), sleep diary completion (ρ=−.75, *P*=.001), and symptom diary completion (ρ=−.53, *P*<.05). Associations between age and adherence with diaries and wearable device were nonsignificant.

**Figure 5:**
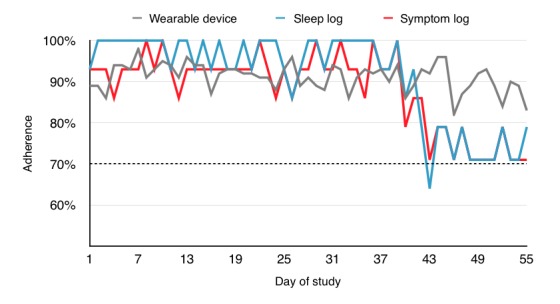
Mean longitudinal adherence to wearable device, sleep, and symptom diaries for all participants over the 8-week study period.

Although adherence to the wearable device remained relatively stable throughout the 8-week study period, sleep and symptom log completion dropped off toward the latter quarter of the study, though remaining mostly above 70% (Figure 5).

### End-of-Study Acceptability and Usability Findings

Overall, responses to the end-of-study questionnaire relating to the *Sleepsight* system and each of its elements were positive (Multimedia Appendix 1). A total of 14 participants completed the end-of-study interview; the majority found the system to be easy to grasp and use, though 1 participant found it complicated. Moreover, 9 participants expressed a wish to continue using the system in its current state and 11 felt that sleep monitoring could be a successful strategy for the early detection of relapse.

All participants found the personalized encouragement text messages to be helpful in improving motivation. In addition, 10 users were motivated by the prospect of receiving devices at the end of the study, and a similar proportion of participants reported frequently using the Fitbit app to view their sleep and physical activity patterns. All participants expressed a wish to keep the study devices after the study had ended. Moreover, 3 participants reported feeling occasionally suspicious toward the technology and worried that their personal information was at times being monitored. However, this did not lead to discontinuation.

Of the 14 participants, 13 reported an inverse association between their sleep and mental well-being, and 12 of the 14 participants reported significant sleep disturbance around the time of their first episode of psychosis or subsequent relapse.

Further themes extracted from the end-of-study interview clustered around 3 major themes: attitudes and beliefs toward the concept of remote sensing for predicting relapse, factors relating to the technology, and factors relating to the users’ illness in interacting with digital technologies.

#### Attitudes and Beliefs Relating to Remote Sensing and Relapse Prediction

All but 1 participant reported experiences of sleep disturbance as an important problem, either during periods of stability or around episodes of acute illness, and welcomed the emphasis on sleep as a possible predictor of deterioration. Many felt that measuring sleep patterns would be an important element of self-management and could imagine how psychological or pharmacological interventions delivered in the early stages of relapse could be therapeutic. Overall, 5 participants commented on how they would have concerns over false alarms—for example, whether sleeping poorly in the absence of deterioration in symptoms would trigger a response. One preferred the terms sleep “tracking” or “logging” rather than “monitoring,” which held connotations of surveillance. Moreover, 4 participants felt that their engagement in the study was influenced by the knowledge that their data were not being analyzed and responded to in real time, and if this were to have been the case, they would have greater levels of engagement.

#### Technology-Related Factors

Half of the users commented that the wearable was at times uncomfortable: the strap would feel tight, which was particularly bothersome in bed. However, most of the users reported initial discomfort, which improved as the study progressed. One participant reported intermittent concern over radiation emerging from the optical heart rate monitor. There were no reports that the sleep metrics were inaccurate; however, 3 participants felt that the Fitbit overestimated step count. A third of participants found value in using the Fitbit for setting and attaining physical activity goals. Charging of the Fitbit or smartphone did not raise any concerns. Two-thirds of participants found that completing the daily symptom diary helped them reflect on mental state and gain greater insight into the links between their symptoms and behaviors, especially sleep. A sense of being “acknowledged” as a specific consequence of using the symptom diary was reported by 3 individuals. Moreover, 2 mentioned how it encouraged family to be involved and became a focus for education. In addition, 6 participants stated that the symptom diary became repetitive and tedious, particularly when the users felt their symptom burden was low, and that if there were no incentive, they would be unlikely to answer the questionnaire on a daily basis. Two users found some questions difficult to understand. A wish to have greater flexibility in the timing of diary completion was raised by 2 users.

#### Illness-Related Factors

A quarter of participants expressed concern that adherence would diminish during relapse. First, users’ cognitive function and therefore the ability to interact with the technology might decrease as symptoms escalate. Second, users may develop suspicion toward the devices, leading to discontinuation. Generally, users felt that the wearable device would be better tolerated in this scenario than the smartphone diaries.

### Comparison of Subjective and Objective Estimates of Rest-Activity

**Figure 6:**
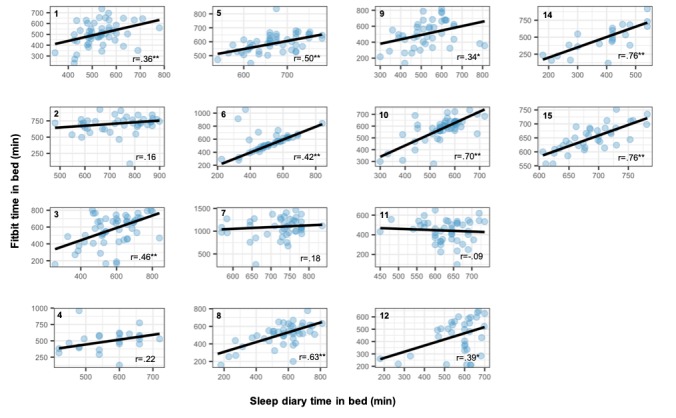
Comparisons of subjectively (sleep diary) and objectively (Fitbit) determined daily time in bed for each participant, fitted with robust bisquare regression to account for outliers. *, ** Significance at the .05, <.01 levels, respectively, for the 2-tailed test.

Correspondence between self-reported (sleep diary) and objectively (Fitbit) determined daily time in bed for all participants showed good overall correlation (ρ=.50, *P*<.001 [2-tailed]). At an individual level, 10 out of 14 patients showed good agreement between subjective and objective measures, reaching the level of statistical significance (Figure 6). Participants 2, 4, 7, and 11 showed poorer agreement between measures, which for participants 4, 7, and 11 are likely to be due to the clustering of sleep diary estimates around the same value, suggesting that these participants gave stereotyped responses that varied little from day to day.

**Figure 7:**
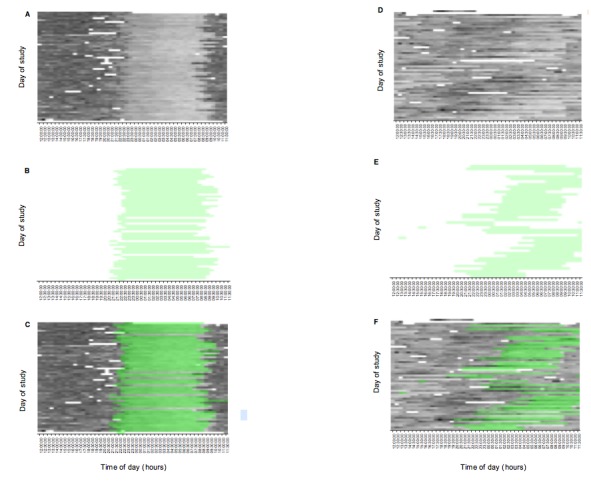
Rest-activity profiles from 2 participants—one with a regular rest-activity profile (A-C) and another with a highly variable, free-running circadian rhythm, not entrained to the day-night cycle (D-F). A and D: heart rate data from the wearable device, with darker shading indicating higher mean heart rate. B and E: subjective sleep times from sleep diary. C and F: subjective sleep times superimposed upon heart rate data (see main text for further details).

More detailed visualization of these data in 2 participants suggests that the Fitbit captured both within and interindividual variability in rest-activity profiles. Figure 7 shows circadian variation in heart rate signal in a middle-aged participant treated with clozapine who led an active social life, with a regular circadian rhythm entrained to the light-dark cycle, and a younger participant, treated with aripiprazole, who spent considerable time in the bedroom playing online computer games. This second case demonstrates a striking free-running non-24-hour circadian rhythm, with no correspondence to the light-dark cycle and progressive delay in sleep-onset**—**an observation that has been previously reported in a single case report [47]. Subjective sleep times collected via the sleep diary, shown in green, suggest a close correspondence between subjective sleep times and objective rest-activity profiles in these participants.

## Discussion

### Principal Findings

However sophisticated the engineering and analytic solutions, the success of digital interventions in improving clinical outcomes will depend crucially on whether they are adopted by patients for long-term use. We collaborated with patients in the design and deployment stages and asked participants with severe mental illness, under the care of secondary level psychiatric services with a range of positive and negative symptom severity, to use wearable and smartphone devices over 2 months in a naturalistic, ecologically valid setting. Our findings suggest that people with schizophrenia are enthusiastic about the concept of sleep disturbance as an indicator of relapse, and they are willing to adapt existing consumer technologies in managing their condition. Consistent with previous research examining smartphone interventions [26,28,31], we observed good levels of adherence with passive monitoring using wearable technologies over the study duration, across a range of ages and symptom severity. Adherence with active monitoring using smartphone diaries tended, however, to drop off toward the latter quarter of the study, suggesting that passive monitoring was more acceptable to users. This raises questions about striking the correct balance when integrating passive and active approaches, each of which have their strengths, and it also raises questions about how motivation with active monitoring can be maintained over time. In particular, users with significant negative symptoms experienced difficulties with completing the sleep and symptom diaries. Although some participants reported mild and intermittent suspicion, paranoia was not a cause of discontinuation. Similarly, there were very few concerns over data security and privacy, which may reflect the efforts that were made to build a trusting, collaborative relationship with participants.

### Motivation and Incentives

The relatively high rates of adherence in this study may be attributable to the incentives that were offered. Though all participants reported this as a motivating factor, the majority also reported intrinsic interest in the system and wished to continue using it after the study ended. Nonetheless, enhancing engagement and motivation by providing users with information that they value is a key challenge for the field. Each user is unique in which elements of the system they find engaging. Some participants may value feedback on physical activity, others on sleep duration, and others on their self-rated symptoms. Adaptable systems that can be tailored to each user may be a solution. Given that this was an observational study, we opted against providing explicit feedback of passive sensor or diary data, due to its liability to influence the underlying behavior we aim to examine; however, this may have reduced levels of adherence. Alternative approaches such as “gamification” [48] are attracting increasing interest.

Furthermore, promoting motivation by providing relatively low-cost mobile communication technologies, which many patients may otherwise be unable to afford, may have the added benefit of enhancing social interaction, communication with the care team, and functioning. In a clinical scenario, should mobile technologies be shown to be effective in reducing relapse, provision of equipment and a mobile data contract would represent a relatively insignificant financial outlay in comparison with the personal, social, and economic costs of relapse [49].

Cost is also an important factor in considering the scalability and reach of digital interventions. The cost of the Fitbit Charge HR was £100, the Moto G smartphone £140, and the mobile data plan £14 per month. The overall cost for consumables per user for the study was, therefore, £270, which equates to approximately just over half the cost of a day on an acute psychiatric ward [50] or the average cost of a monthly dose of paliperidone long-acting injectable antipsychotic medication [51]. As affordability and ownership of digital technologies grows in populations with serious mental illness [28,29,52], this approach has the potential to address the disparities in health care provision in underserved populations with serious mental illness globally [53].

### Consumer Devices in Clinical Research

The use of consumer wearable devices in clinical contexts raises several important considerations. These devices are marketed as products that claim to enhance fitness and well-being, often supported by bold but unsubstantiated claims. Although some validation data are published from healthy populations showing acceptable agreement between consumer devices to actigraphy for some physical activity measures [54] and sleep [55] and with ECG for heart rate [56,57], no such data are available for populations with psychosis. Do these devices, therefore, have a role as tools for clinical prediction?

We suggest that they do, depending on the question being asked [58]. Our goal is not to draw conclusions about sleep parameters (eg, total sleep time, sleep efficiency) per se, for which the use of unvalidated devices would be inappropriate. Rather, our objective is to ask whether changes in longitudinal rest-activity patterns at the within-person level, captured using wearable device and smartphone sensors, predict deterioration in clinical status. The measurement error of these devices in healthy populations is comparable with that of actigraphy [54,55], and given the high level of noise inherent in free-living conditions, we hypothesize that should a signal exist, both consumer and research devices should be able to capture it. Comparison of subjective and objective estimates (Figure 6) and visualization of wearable data (Figure 7) suggest that consumer devices capture both inter- and within-individual variability in rest-activity patterns, and our subsequent work will primarily use within-person analyses to evaluate the association between these predictors and mental state, making calibration and interdevice reliability less of an issue.

Though we welcome further validation studies of consumer against gold standard devices, consumer devices follow a rapid product cycle, meaning they are rapidly superseded by newer models. Deciding on which device to study, and in which clinical groups, is therefore challenging. Another issue is the closed nature of these devices, due to reasons of intellectual property, manufacturers do not publish the algorithms through which activity and sleep outputs are calculated, and the researcher generally lacks control over the implementation of algorithm updates during the course of a study. In addition, preprocessing of data on the consumer device limits their granularity to minute-level data, at best. In turn, this might constrain the choice of subsequent analysis, for example, it is uncertain whether nonlinear patterns of motor activity [59,60] can be extracted from these data.

In navigating these challenges, greater collaboration between medical and consumer device manufacturers (each of whom operate a fundamentally different business model), researchers, and consumers will be necessary for driving innovation in this area. It is worth reiterating that research grade, wearable accelerometers that are acceptable for long-term use do not currently exist, and further research is urgently needed to evaluate the reliability and validity of consumer wearable and mobile devices as clinical tools [61]. Regardless of its accuracy, the clinical utility of a device will be limited if adherence is poor.

### Limitations

This small pilot study was designed primarily to evaluate feasibility and acceptability in patients in remission, and at this stage, we are unable to draw any conclusions about its potential for identifying early markers of relapse or offer evidence of the validity of the signal from the wearable device. Our supplementary trial suggested that the absence of HR signal is highly correlated to nonwear; however, movement of the device may activate the HR sensor, and there remains the possibility that spurious readings were produced while the device is removed from the wrist. Participants were in a relatively stable phase of illness, and the influence of worsening psychopathology on adherence was not examined directly. Future studies should aim to test adherence over longer periods, in more unstable populations such as in the period following hospitalization [62]. Although a user group involving young people was consulted in the development phase, the field study did not include younger adults under the care of early intervention services. This group is particularly likely to benefit from early intervention strategies and also engage well with digital interventions. The name of the study, which emphasized sleep in psychosis, may have led to a bias toward the recruitment of participants who experienced difficulties with sleep, which may have improved acceptability. However, this also illustrates the importance of designing interventions that address clinical problems, which patients perceive to be important, and framing them as such. We did not test the acceptability of collecting GPS location data, which recent studies suggest may be a useful feature in predicting clinical status in a bipolar disorder [63]. As rates of smartphone ownership among people with mental illness grow, the incentive value of providing patients with smartphones may diminish.

### Conclusions and Future Directions

We found enthusiasm and high levels of engagement with passive monitoring in people with schizophrenia, which exceeded our acceptability thresholds, across a range of ages, symptom dimensions, and severity. However, mHealth technologies are unlikely to be acceptable to all patients, and it will, therefore, be important to further understand which groups are most likely to engage with and benefit from these interventions, while maximizing their reach by developing adaptable, tailored tools. An inherent property of participatory interventions is that they are likely to improve participants’ outcomes and relapse risk, for example, by encouraging the user to reflect on their symptoms and monitor variables such as their sleep [64]. This has important implications in the design of future mHealth research and argues for studies that detect the improvement in outcomes in a randomized controlled design rather than a purely observational design.

A further under-researched area is how the adoption of digital interventions by health care professionals can be facilitated [65] such that clinical decision making is enhanced, rather than being perceived as an additional burden to an already busy schedule. Listening to the needs of clinicians in the co-design of technologies will be integral to their success, as will secure integration with existing medical record systems.

Extracting clinically meaningful information in real time from high-volume, multidimensional data represents another major challenge [66]. Although our interest focuses on circadian rest-activity profiles, combining additional sensor variables such as the ultradian organization of motor activity [12,59] and speech analysis [67] is likely to improve precision. The signature and optimal thresholds for relapse are likely to be different for each patient, and the inputs, sensitivity, and specificity of algorithms will, therefore, need to be tuned to each individual.

A long-term ambition of this approach is to enhance clinical care by identifying early signs of deterioration and thereby facilitate self-management and preventative psychological or pharmacological intervention. As research in this area continues to advance, future efforts should focus upon how clinicians, patients, technologists, and the industry can work together effectively to maximize the clinical utility, validity, and implementation of these novel tools.

## Appendix

Multimedia Appendix 1 Responses to end-of-study questionnaire; validity of heart rate signal for adherence.

## Abbreviations

API: application programming interface
GPS: Global Positioning System
PANSS: Positive and Negative Syndrome Scale
