# Supplementary material for: Capturing Rest-Activity Profiles in Schizophrenia Using Wearable and Mobile Technologies: Development, Implementation, Feasibility, and Acceptability of a Remote Monitoring Platform
Source: JMIR Mhealth Uhealth. 2018 Oct 30;6(10):e188. doi: 10.2196/mhealth.8292 (PMC6234334; doi:10.2196/mhealth.8292)
Supplement: Multimedia Appendix 1 [file mhealth_v6i10e188_app1.pdf]

Question	Agree	Neutral	Disagree
Sleepsight system as a whole			
Overall, I am satisfied with how easy it is to use Sleepsight	14	0	0
It was easy to learn to use Sleepsight	13	1	0
I found Sleepsight to be very complicated	1	1	12
The information provided for Sleepsight was easy to understand	11	3	0
How things appeared on the screen was clear	14	0	0
Sleepsight helped me manage my symptoms	6	6	2
If I have access to Sleepsight, I would use it	9	3	2
Monitoring sleep might work as a relapse detection tool	11	3	0
Motivation and incentives			
I felt motivated to use Sleepsight regularly	13	1	0
Knowing that I would receive the devices at the end of the study motivated me to use them	10	3	1
I would have been just as motivated if I used my own phone	6	4	4
I will continue to use Sleepsight once the study has ended	9	2	3
I am not interested in keeping the smartphone	0	0	14
I am not interested in keeping the Fitbit	1	0	13
I found the weekly encouragement texts helpful	14	0	0
I found the weekly encouragement texts annoying	0	0	14
Sleepsight app			
Generally, I completed the questionnaire at the time of the alarm	9	1	4
The sleep and symptom questionnaire was quick to complete	14	0	0
The Sleepsight app worked smoothly	13	0	1
The Sleepsight app was intuitive to use	12	2	0
I looked forward to filling in my questionnaire	6	7	1
Smartphone			
I remembered to keep my smartphone charged at all times	14	0	0
I took my smartphone with me when I left the house	12	2	0
I regularly used my smartphone for making calls	4	4	6
I regularly used my smartphone for sending text messages	3	3	8
I will continue to use the smartphone after the study has ended	12	1	1
Fitbit			
I found wearing the Fitbit uncomfortable	3	3	8
I enjoyed wearing the Fitbit	13	1	0
I remembered to keep my Fitbit charged at all times	13	1	0
I felt self-conscious wearing the Fitbit	1	3	10
The Fitbit looked stylish	13	1	0
People I meet are interested in the Fitbit	8	4	2
Charging the Fitbit was difficult	2	3	9
I generally remembered to put my Fitbit back on after bathing	13	1	0
The Fitbit battery reminders were helpful	13	0	1
I often used the Fitbit app to look at my activity patterns	10	2	2
Risks and safety			
Using Sleepsight sometimes made me suspicious	1	3	10
I worried that my personal information was being monitored	3	1	10
I felt I was put at risk through using Sleepsight	0	1	13

Table 3, supplementary material: responses to end of study questionnaire.

Validity of HR signal for adherence

The validity of the HR signal as a proxy for wear and non-wear of the Fitbit Charge HR was tested in two individuals, by wearing the device for 60 minutes and removing it for 60 minutes (repeated twice); then 30 minutes on/off (repeated twice); then 15 mins on/off (repeated twice).

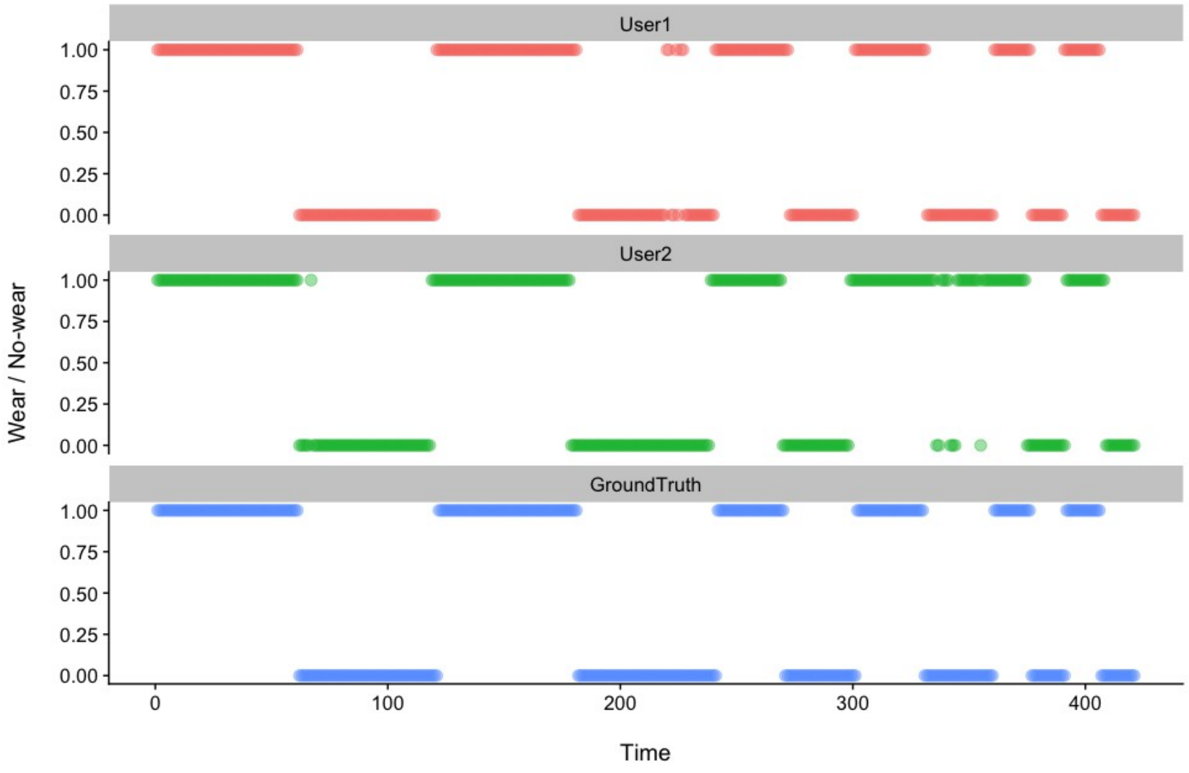

Figure 6. Validity of HR as a proxy for wear/non-wear of wearable device. Ground truth: wear = 1 and non-wear =0; User 1 and User 2: HR signal present = 1; HR signal absent = 0. Time, minutes.

Sensitivity, specificity, accuracy and cohen's kappa was high for both users, suggesting the presence or absence of HR signal closely mirrors the wear or non-wear of the device.

Tester 1:
Sensitivity = 0.94
Specificity = 1.00
Accuracy = 0.97
Kappa = 0.94
Confusion matrix, user 1:

	Non-wear, minutes	Wear, minutes
HR signal absent, minutes	199 (true negative)	0 (false negative)
HR signal present, minutes	12 (false positive)	210 (true positive)

Tester 2:
Sensitivity = 0.82
Specificity = 0.97
Accuracy = 0.90

Kappa = 0.80
Confusion matrix, user 2:

	Non-wear, minutes	Wear, minutes
HR signal absent, minutes	175 (true negative)	6 (false negative)
HR signal present, minutes	36 (false positive)	204 (true positive)
